# Respiratory Anatomy and Physiology of Reptiles

**DOI:** 10.3390/ani16091396

**Published:** 2026-05-02

**Authors:** Javier G. Nevarez

**Affiliations:** Department of Veterinary Clinical Sciences, Louisiana State University School of Veterinary Medicine, Baton Rouge, LA 70803, USA; jnevare@lsu.edu

**Keywords:** reptile, lungs, anatomy, physiology

## Abstract

Reptiles have varied lung anatomy with three main lung types: unicameral, transitional, and multicameral. Crocodilians have a well-developed bronchial tree, while all other species have either a limited number of bronchi or no bronchi at all. The primary gas exchange structures are the faveoli and ediculae, which can have either homogeneous or heterogeneous distribution within the lung parenchyma. The physiology of reptile respiration is also complex, varied, and at times contradictory across species. A basic understanding of anatomy and physiology is essential for proper diagnostic and therapeutic approaches to reptile patients. Therefore, a paradigm shift is needed to start thinking of the anatomy and physiology of individual species rather than believing that a single concept applies to all reptiles equally. As such, future research should aim to duplicate concepts across the more common species encountered in clinical practice.

## Simple Summary

Reptiles have far more variability in their respiratory anatomy than what is found in other taxa like birds and mammals. There are also important physiological adaptations that allow reptiles to improve the efficiency of oxygen exchange, even in their lungs, with a simple structure. This article will provide an in-depth review of lung anatomy and physiology with their relevant clinical implications.

## 1. Anatomy

The upper respiratory tract is composed of the nares, nasal passages, and choana. While the nares of most reptiles are simple openings to allow passage of air, some species have unique adaptations. Marine iguanas (*Amblyrhynchus cristatus*) have a slight extension of tissue beyond the nasal opening to help expel salt from their salt glands. The most complex nares are found in crocodilians. They possess musculature, the constrictor naris and dilator naris, that allow voluntary closing and opening of the nares, respectively [1]. This adaptation is likely because crocodilians are the only reptile group to have a secondary bony palate (often termed the hard palate in mammals) and pharyngeal choana as opposed to a choanal opening in the oral cavity. They also have a palatal valve that further seals the pharyngeal cavity when underwater. This valve blocks the flow of water that would otherwise go directly into the glottis and esophagus. The muscular nares, together with the ability to seal the nasal passages through engorgement of the vascular cavernous tissue, allow crocodilians to prevent the intrusion of water through the nasal passages when underwater. Nasal conchae are simpler structures than in mammals and are absent in chelonians. Instead, the nasal cavities of chelonians have a series of non-bony diverticula.

A vomeronasal organ (a.k.a. Jacobson’s organ) has been described in all reptile groups but is not present in adult crocodilians. In squamates, the vomeronasal organ is separate from the nasal cavity and is in the most cranial aspect of the maxilla, with openings into the oral cavity that connect to the accessory olfactory bulb. Its main function is the detection of prey and recognition of conspecific smells that may arise from various musk glands. The most obvious example of the functionality of the vomeronasal organ can be found in snakes and varanid lizards. These reptiles use their forked tongues to gather olfactory cues from the environment, followed by insertion of the tips of the tongue into the openings of the vomeronasal organ so the information can be processed through the accessory olfactory bulb. There is a scarcity of information about clinical diseases associated with the vomeronasal organ in reptiles. One study demonstrated a behavioral response difference in North American vipers exposed to a kingsnake [2]. Most viper species had a decreased defense posture after the vomeronasal openings were sutured closed. Normal defensive behaviors were observed before suturing and once again after sutures were removed, providing evidence of the functional role of the vomeronasal organ. Therefore, an alteration in olfaction must be considered a possibility in cases of stomatitis or upper respiratory tract disease affecting the openings of the vomeronasal organ. This theoretically could lead to perceived anorexia or altered behaviors due to a decreased olfactory ability.

**Figure 1:**
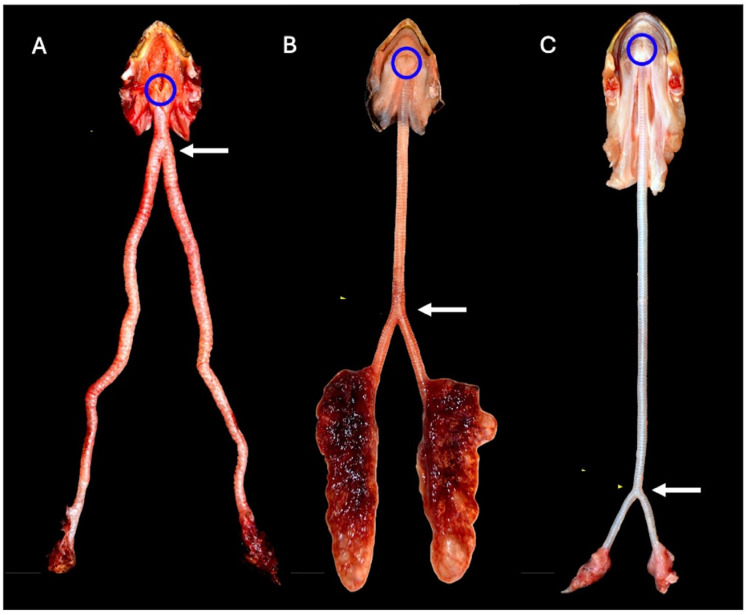
Differences in the location of the glottis and tracheal lengths in three chelonian species: (**A**) Marginated tortoise (*Testudo marginata*; Cryptodira); (**B**) Caspian turtle (*Mauremys caspica*; Cryptodira); and (**C**) Northern snake-necked turtle (*Chelodina rugosa*; Pleurodira). The blue circle denotes the location of the glottis. The white arrows denote the location of the bifurcation of the trachea, which is more caudal in the Pleurodira. Image adapted from Habova M, Pyszko M, Horak O, Cermakova E, Paral V. “Differences in the anatomy of the lower respiratory tract in selected species of the order Testudines”. *Vet Med* (Praha). 2022 Feb 15;67(2):78–86. doi: 10.17221/64/2021-VETMED [4].

**Figure 2:**
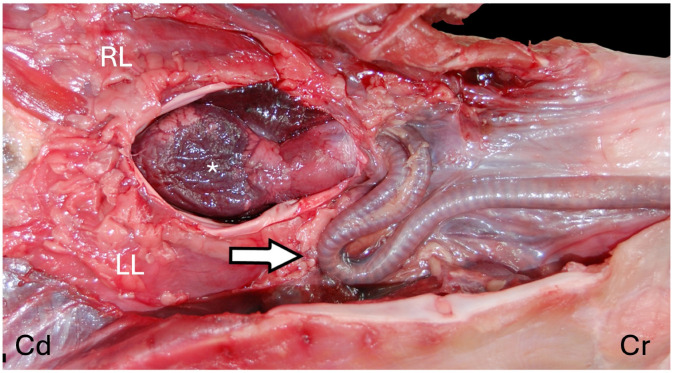
Tracheal loop (white arrow) in a Nile crocodile (*Crocodylus niloticus*). Cranial (Cr), caudal (Cd), heart (*), left liver lobe (LL), and right liver lobe (RL). Image adapted from Schachner ER, Hutchinson JR, Farmer C. “Pulmonary anatomy in the Nile crocodile and the evolution of unidirectional airflow in Archosauria”. *PeerJ*. 2013 Mar 26;1:e60. doi: 10.7717/peerj.60 [6].

**Figure 3:**
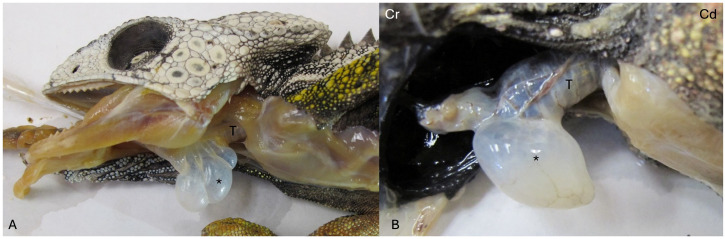
Gular pouch in chameleons. (**A**) Spiny chameleon (*Furcifer verrucosus*) has multiple gular pouches (*) between the larynx and trachea (T). (**B**) Meller’s chameleon (*Trioceros melleri*) has singular gular pouch (*) cranioventral to the trachea (T). Cranial (Cr) and caudal (Cd). Image courtesy of Dr. Steve Huskey.

The lower respiratory tract is composed of the trachea, bronchi, and lungs. Squamates (snakes and lizards) have incomplete tracheal rings, while chelonians and most crocodilians have complete tracheal rings. Incomplete tracheal rings are reported in the broad-snouted caiman (*Caiman latirostris*) and yacare caiman (*Caiman yacare*) [3]. In practice, endotracheal tube cuffs should not be used or inflated in those species with complete tracheal rings to avoid trauma. Even in snakes and lizards, use/inflation of the cuff should be done gently, as the tracheal tissue may be more delicate than in mammals. A high degree of variation in tracheal length and the split into a left and right bronchus have been described amongst various chelonian species [4] (Figure 1). In some crocodilians, there is also variability in the trachea’s location within the neck and its association with the esophagus [5]. In addition, crocodiles have been shown to have a tracheal loop (Figure 2) in the distal trachea. This loop may be present in juveniles or develop with age, depending on the species [5,6]. A tracheal loop is absent in the American alligator (*Alligator mississippiensis*). There appears to be less tracheal variability amongst snakes and lizards. However, an important exception is found in Chamaeleonidae, in which the presence of a gular pouch associated with the trachea has been described [7]. The gular pouch extends either from the location where the larynx and trachea meet or from between the tracheal rings themselves [7] (Figure 3). It is thought to be involved in the production of sounds in chameleons. Differences in size and presence of the gular pouch are also described across different species [7]. From a clinical perspective, the gular pouch often makes endotracheal intubation difficult as the tube may get lodged in the pouch. One must be careful not to be too forceful and tear the pouch. Also, the tube must be advanced beyond the pouch to ensure proper ventilation.

**Figure 4:**
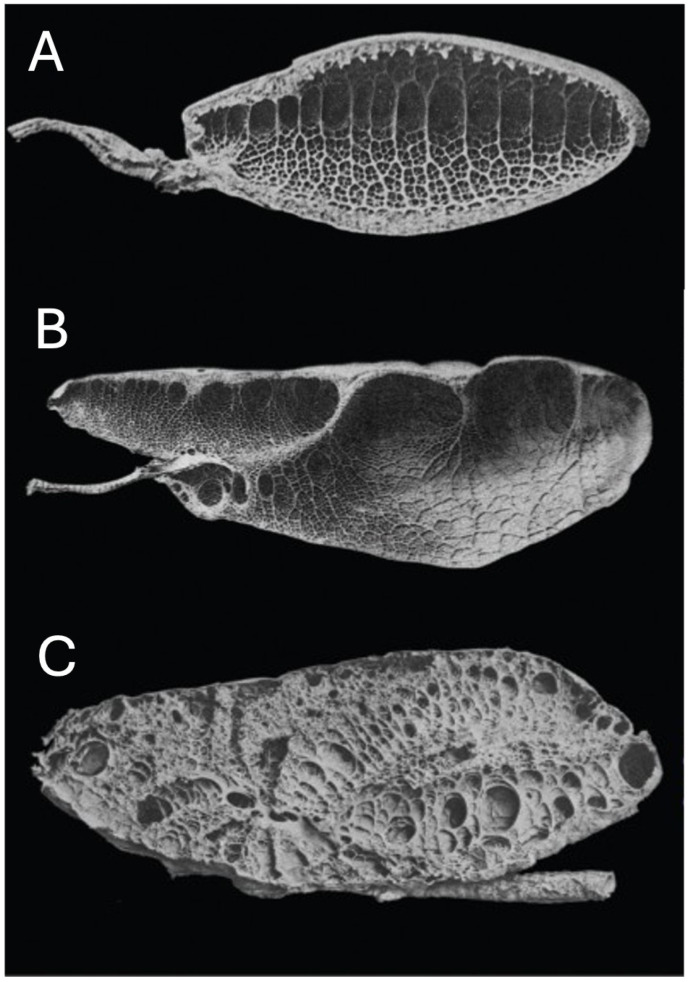
Longitudinal lung sections. (**A**) Unicameral lung from a gecko. (**B**) Paucicameral lungs from a green iguana (*Iguana iguana*). (**C**) Multicameral lung from a crocodilian. Adapted from Schachner ER, Cieri RL, Butler JP, Farmer CG. “Unidirectional pulmonary airflow patterns in the savannah monitor lizard”. *Nature*. 2014 Feb 20;506(7488):367–70. doi: 10.1038/nature12871 [9].

**Figure 5:**
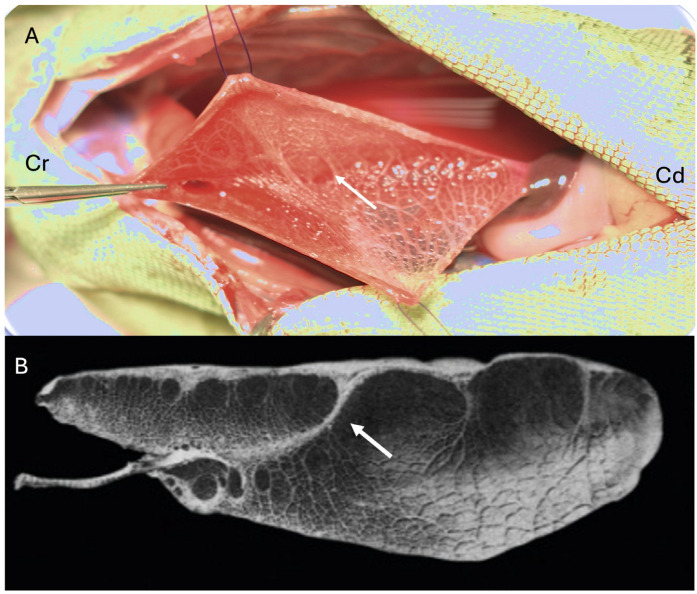
(**A**) Dissection of a green iguana (*Iguana iguana*) showing the exposed lumen of the paucicameral lung. (**B**) Gray-scale image of a green iguana lung. Cranial (Cr) and caudal (Cd). White arrows show the parenchymal invagination that creates partial separation of the lumen. Dissection image courtesy of Javier G. Nevarez. Greyscale image adapted from Schachner ER, Cieri RL, Butler JP, Farmer CG. “Unidirectional pulmonary airflow patterns in the savannah monitor lizard”. *Nature*. 2014 Feb 20;506(7488):367–70. doi: 10.1038/nature12871 [9].

**Figure 6:**
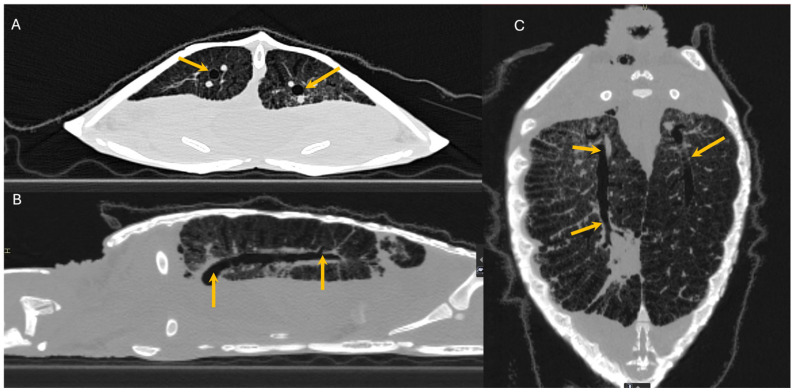
CT image series of Kemp’s ridley (*Lepidochelys kempii*) turtle. (**A**) Cross-sectional view. (**B**) Sagittal view. (**C**) Coronal view. Orange arrows show the primary bronchus through the multicameral lung. Image courtesy of Javier G. Nevarez.

Reptiles have one of three main lung types based on the complexity of the parenchymal tissue: unicameral, paucicameral (transitional), or multicameral. Unicameral lungs have a simple structure with a common lumen without any internal divisions and lack a mammalian-type bronchial tree (Figure 4). Each lung is a single chamber. Paucicameral lungs, also referred to as transitional lungs, have a similar structure to the unicameral but with partial divisions of the lumen through invagination of the parenchymal tissue, creating a small number of chambers (Figure 4 and Figure 5). A bronchial tree is also absent. Multicameral lungs are the most complex, with a structure that more closely resembles the lungs of birds and mammals. Multicameral lungs have thick parenchyma with multiple chambers. Crocodilians have multicameral lungs with a well-developed bronchial tree, like what may be expected in mammals (Figure 4). Chelonians have multicameral lungs but lack a bronchial tree. Instead, chelonians possess a primary bronchus that runs along the long axis of the lung without splitting into any smaller branches (Figure 6) [8]. This primary bronchus has openings along its length (e.g., ostia) that allow air to move into various chambers [8]. Varanid lizards also have multicameral lungs with a primary bronchus with ostia spanning the length of the lung, but some smaller bronchi arising to feed chambers can be found [9]. However, a bronchial tree per se is lacking. From an anatomical and physiological standpoint, it is important to be familiar with the various structures that compose the lung. These can be divided into supraparenchymal, parenchymal, and subparenchymal structures.

### 1.1. Supraparenchymal Structures

The supraparenchymal structures include the intrapulmonary airway, lumen, chamber, lobe, and niche [10]. The intrapulmonary airway can be either the trachea itself, a bronchus, or a duct, depending on the lung type and species. In many species with unicameral lungs, such as snakes, the trachea opens into the lumen of the lung without transitioning into a bronchus. In other species, the trachea splits into a right and left bronchus before entering the lung. In unicameral and paucicameral lungs, there is often a single cartilaginous bronchus that opens into the lumen or chamber [11]. If the bronchus loses its cartilaginous structure as it enters the lung, then it is classified as a duct [11]. In multicameral lungs of crocodilians, there is an extensive bronchial tree. Varanids and chelonians have multicameral lungs; however, rather than a bronchial tree, they have a primary bronchus that runs the entire length of the lung. Smaller bronchi may be found in some varanids [9]. Ciliated, goblet, and/or secretory cells can be found lining the intrapulmonary airway [11]. The next structure is the lumen, which is simply the air-filled space(s) of the lung. The lumen is within or surrounded by chambers, lobes, and niches. The chambers are primary subdivisions of the lumen, mostly in paucicameral and multicameral lungs. In paucicameral lungs, the chambers are formed by extensions of the parenchymal tissue, creating partial divisions and opening into the central lumen [10]. In multicameral lungs, the intrapulmonary airway connects directly to the chambers. The lobes are further subdivisions of the chambers and connect with the central lumen of the chamber or the lumen itself, but do not have a direct connection to the intrapulmonary airway [10]. The niche is the smallest of the supraparenchymal structures and is an invagination of the parenchymal tissue arising from the chamber or lobe.

### 1.2. Parenchymal Structures

**Figure 7:**
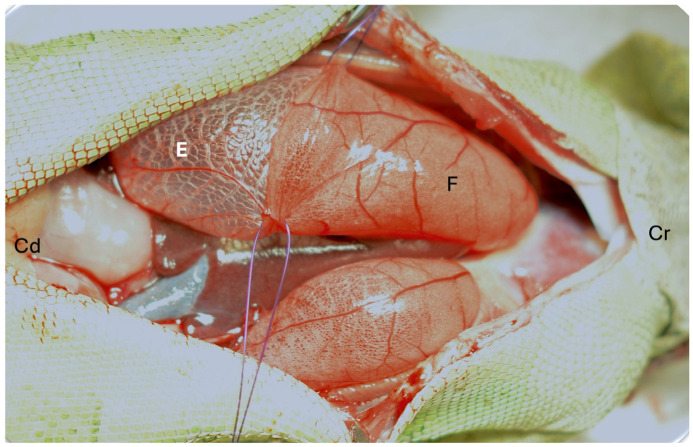
Inflated lungs of a green iguana (*Iguana iguana*) showing the heterogeneous nature of the parenchyma. Cranial (Cr), caudal (Cd), faveolar portion (F), and edicular portion (E). The trabeculae are the grossly visible lines in the edicular portion. Image courtesy of Javier G. Nevarez.

There are three parenchymal structures: faveoli, ediculae, and trabeculae. The faveoli and ediculae are analogous to the alveoli of mammals. Structurally, the faveoli are narrow and deep openings within the parenchyma. The faveoli form a thick mass of parenchymal tissue, are well vascularized, and look similar to what is expected of alveolar tissue in mammals. The ediculae are wide and shallow areas on the parenchyma. Ediculae are less vascularized and form the thin layer of parenchymal tissue. When present, the air sacs in reptiles are often composed of edicular tissue. The faveoli and ediculae are dead-end spaces of the parenchymal tissue. The distribution of faveoli and ediculae can be homogeneous or heterogeneous. Unicameral and paucicameral lungs are often heterogeneous, with the cranial portion being faveolar and transitioning to a thinner edicular portion caudally (Figure 7). Snakes and chameleons are better known for the “air sac” caudal portion of their lungs. These portions can be a transition from the faveolar to the edicular or trabecular lung. The air sac may also have aparenchymal regions. Even lizards, such as green iguanas (*Iguana iguana*) and bearded dragons (*Pogona vitticeps*), have a significant portion of their caudal lung that is edicular but is often not appreciated until the lung is fully inflated (Figure 6), unlike snakes and chameleons, in which the edicular/air sac portion is grossly visible at all stages of respiration.

The trabeculae are made up of elastic tissue and smooth muscle. They are the scaffolding that supports the parenchymal tissue and outlines the openings of the faveoli and ediculae. Trabeculae are grossly visible on fresh tissue, radiographs, and during coelioscopy or pulmonoscopy (Figure 7). Trabeculae may be lined with clearance epithelium. Some may also be lined with capillaries and contain smooth muscle and neuroepithelial bodies [11,12]. During times of hypoxia, serotonin secretion leads to contraction of the smooth muscles, causing the trabeculae to change shape. This, in turn, allows the faveoli and/or ediculae to change shape and mobilize air, helping with the diffusion of gases [11,13]. Perry also proposed the possibility that contractions of the trabeculae may lead to exposure of the capillaries in times of hypoxia [11]. This theory, if proven, would make the trabeculae part of the gas exchange structures together with the faveoli and ediculae, although admittedly to a much lesser degree.

### 1.3. Subparenchymal Structures

Trabeculae are also a subparenchymal structure, together with the septa. The septa are composed of collagen, connective tissue, and smooth muscle [11] that connect the trabeculae with each other and with the subpleural lung. The septa form the entrance to the faveoli and ediculae. There are different levels of septa progressively getting smaller and thinner as they approach the wall of the lung. The septa also have capillary nets of varying degrees.

With knowledge of the lung structures, one can visualize the flow of air through the reptile lung. Air travels through the nares, nasal passages, and choana before reaching the glottis. It then enters the trachea and flows into the right and left bronchus before entering the intrapulmonary airway, which may be a simple duct (most squamates), a single primary bronchus (chelonians and varanids), or a bronchial tree (crocodilians). Air then flows into the lumen, chambers, lobes, and niches before entering the faveoli or ediculae. In homogeneous lungs, there is an even distribution of faveoli or ediculae, while heterogeneous lungs are typically faveolar and transition to an edicular portion caudally. The faveolar lung represents a more dense and vascularized parenchyma than the edicular lung.

## 2. Lung Anatomy by Taxonomic Group

### 2.1. Rhynchocephalia

This order includes a single species, *Sphenodon punctatus*, the tuatara. Tuataras have peculiar and unique anatomy and physiology within reptiles. Their lungs are described as unicameral, homogeneous, and edicular. They lack bronchi, and the hilus is located near the apex of the lung [11]. Unfortunately, there is limited information on this species compared to other reptiles.

### 2.2. Ophidia

Ophidians have unicameral heterogeneous lungs that transition from faveolar cranially to edicular caudally. There is a marked transition between the faveolar and edicular tissue, which is commonly called an air sac. Most ophidians have a single right lung, but a left lung can also be found in boas, pythons, and anacondas. While the left lung is often described as reduced or vestigial, in the author’s experience, the difference in length between the right and left lung in boids is primarily due to the air sac rather than the faveolar portion, which tends to be of similar length bilaterally. In general, the air sac ends near the gallbladder in terrestrial species but may extend caudally near the cloaca in aquatic species as part of their adaptation for buoyancy. In arboreal and climbing snake species, the faveolar portion of the lungs comprises less than 20% of the total body length, while in some aquatic species, it can extend the whole length of the body [12]. In viperids and other snake species, a faveolar portion of the lung can be found anterior to the heart and extending to the neck region. This portion of the lung is termed the tracheal lung in these species [14]. Marine snakes have additional anatomical and physiological features that make them unique amongst ophidians, such as cutaneous gas exchange [15].

### 2.3. Testudines

Chelonians have an unbranched intrapulmonary bronchus reinforced with cartilage that runs the length of a multichambered lung. The number of chambers will vary across species. There is either a post-pulmonary septum or mesopneumonium that suspends the lungs from the dorsal body wall [8]. The lungs are covered by the peritoneum and septum. Muscles from the front and hind limbs attach to the cranioventral and caudoventral aspects of the lungs to help facilitate lung movement for inspiration and expiration [8], hence why some slight limb movement may be observed during respiration. This also aids in the performance of cardiovascular resuscitation, by which the limbs can be synchronously pushed and pulled to help move air within the lungs. The primary bronchus arrangement makes it more difficult for any exudate or infectious material to be transported from the parenchyma.

### 2.4. Sauria

Lizards have the highest variability in lung anatomy across any reptilian taxa. Therefore, we present a general overview of the different families, but with the knowledge that exceptions may always exist. The length of the lungs can vary, with some species having a longer left or right lung. Lungs can be homogeneous, faveolar or edicular, or heterogeneous. A post-pulmonary septum has been described in varanids and teiid lizards. Surgical transection of this septum does not appear to affect ventilation at rest but does compromise it under exercise conditions, leading to a decreased tidal volume and altered ventilation patterns [16]. Iguanidae and Agamidae have transitional, heterogeneous lungs. The Gekkonidae have a wide diversity of lung anatomy, but in general are unicameral, faveolar to edicular, or trabecular. Varanidae have multichambered, heterogeneous lungs, with the left often being longer. A post-pulmonary septum attaches laterally to the body wall, creating separation between the intestinal tract, heart, and lungs [10]. In Helodermatidae, the lungs are multichambered with a larger right lung. A post-pulmonary septum covers the ventral aspect of the lungs. The septum runs from the caudal pericardium to the nephric fold and attaches to the lateral liver, creating separation between the intestines, lungs, and heart, like in varanids [10]. Chamaeleonidae have mostly transitional lungs that are primarily edicular, ending in air sacs without trabeculae [17]. Different levels of internal divisions created by septa may be found [17].

### 2.5. Crocodilians

**Figure 8:**
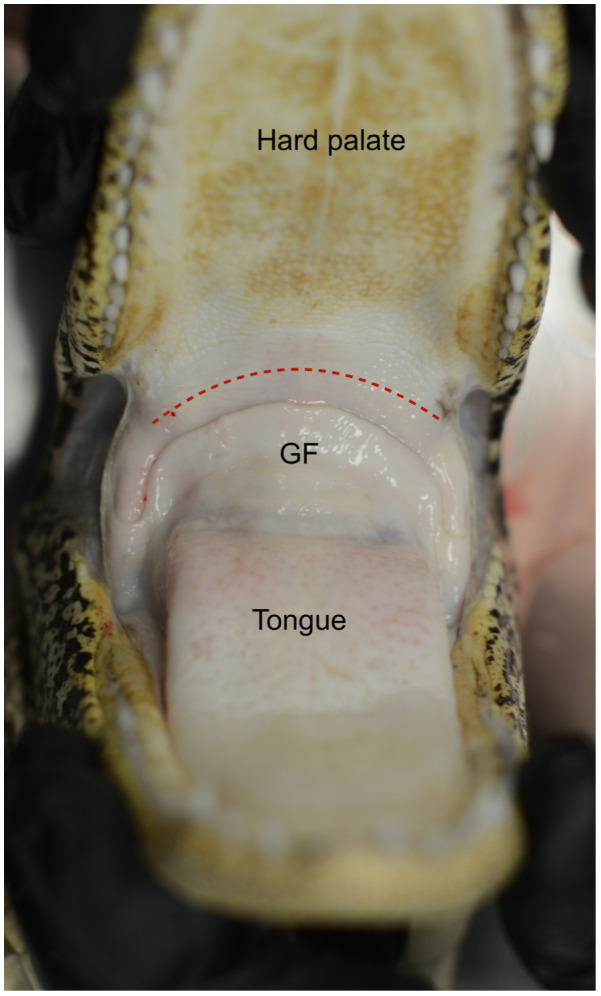
Oral cavity of an American alligator (*Alligator mississippiensis*). The gular valve is composed of the velum palati dorsally (red dotted line) and the gular fold ventrally (GF). Photo courtesy of Javier G. Nevarez.

**Figure 9:**
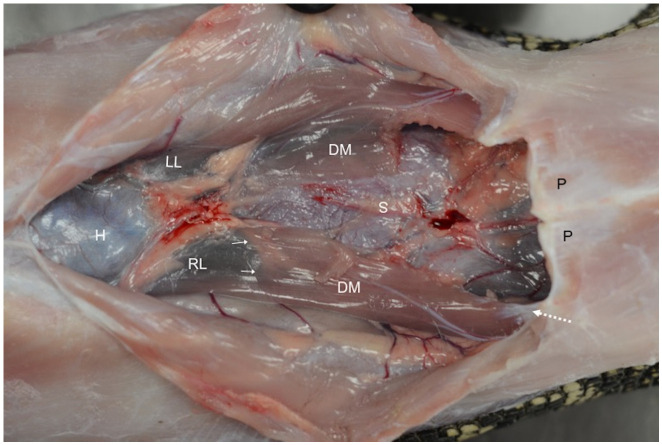
Coelomic cavity of an American alligator (*Alligator mississippiensis*). The diaphragmaticus muscle (DM) has been partially resected during dissection, but its origin from the ischia and posterior gastralia (white dotted arrow) and insertion at the connective tissue on the caudal aspect of the liver (white arrows) are intact. Heart (H), stomach (S), right liver (RL), left liver (LL), and pubis bone (P). Photo courtesy of Javier G. Nevarez.

Crocodilians have multicameral lungs with the most elaborate bronchial tree in reptiles. They have a secondary bony palate, and the gular valve separates the oral and pharyngeal cavities (Figure 8). One of the most unique features of crocodilians is the presence of a diaphragmaticus muscle. This muscle has a horizontal orientation in the coelomic cavity as it originates at the ischia and posterior gastralia (i.e., free-floating ribs) and inserts at the caudal liver lobes (Figure 9). Contraction leads to caudal movement of the liver, which allows for expansion of the lungs during inspiration, while relaxation allows the liver to move cranially to aid in exhalation. For this reason, the mechanism is colloquially referred to as the hepatic piston. Transection of the diaphragmaticus muscle led to shorter dive times in American alligators, but no further clinical effects were noted [18].

## 3. Clinical Implications

**Figure 10:**
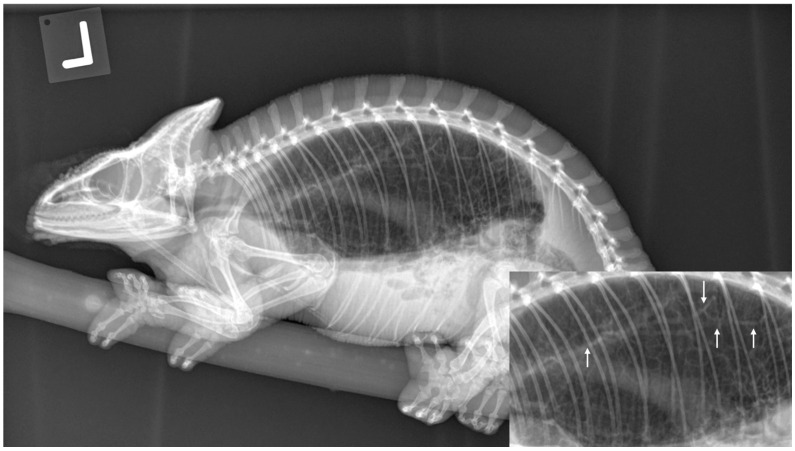
Lateral radiograph of a Parson’s chameleon (*Calumma parsonii*). Insert shows the fine detail of the trabeculae (white arrows) that can be observed radiographically. This normal trabecular pattern can be misinterpreted as a bronchial pattern. Image courtesy of Javier G. Nevarez.

**Figure 11:**
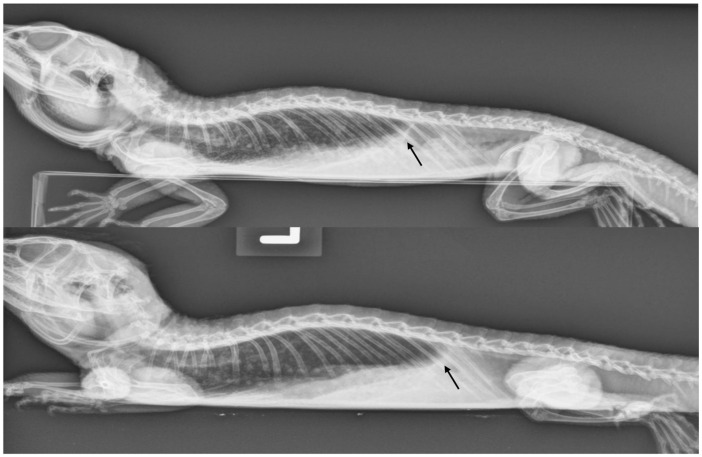
Lateral radiograph of a bearded dragon (*Pogona vitticeps*) diagnosed with bacterial pneumonia before (**top image**) and after treatment (**bottom image**), taken 40 days apart. A difference in the inflation of the lungs is shown with the black arrows. In the post-treatment image, the lungs are more inflated and occupy a larger portion of the coelomic cavity. Image courtesy of Javier G. Nevarez.

**Figure 12:**
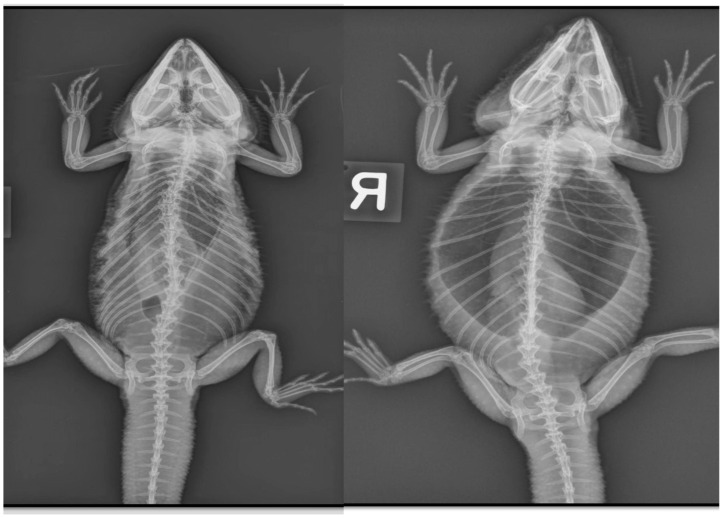
Dorsoventral radiograph of a bearded dragon (*Pogona vitticeps*) diagnosed with bacterial pneumonia before (**left image**) and after treatment (**right image**), taken 40 days apart. In the post-treatment image, the lungs are more inflated and occupy a larger portion of the coelomic cavity. Both images were obtained with the animal at the relaxation phase (apnea phase) of respiration. Image courtesy of Javier G. Nevarez.

There are significant clinical implications for understanding reptilian lung anatomy as it relates to the diagnosis and treatment of respiratory diseases. Radiographs are often the first diagnostic tool utilized. Radiographs have been shown to aid in the evaluation of cold-stunned Kemp’s ridley sea turtles (*Lepidochelys kempii*) [19]. However, radiographs are often not the most sensitive tool for the diagnosis of respiratory diseases in reptiles. Computed tomography (CT scan) was shown to be superior to radiographs in the diagnosis and monitoring of response to therapy of bacterial pneumonia in Indian pythons (*Python molurus*) [20]. The diagnostic value of radiographs is perhaps more of a limitation in reptiles with unicameral or transitional lungs than in those with multicameral lungs. Additional studies are needed to evaluate the sensitivity of radiographs across lung types. CT scans can provide earlier diagnosis and better localization of respiratory disease. Specifically, CT scans can help localize the disease to either the faveolar or the edicular portion of the lung. This information can then help guide the selection of the therapeutic approach as well as inform expectations for recovery. In chelonians and varanids, the arrangement of a multicameral lung with either an unbranched or minimally branched primary bronchus means the treatment of pneumonias and other respiratory diseases may be more challenging. In these species, it is more difficult for secretions to drain from the parenchymal tissue to the chambers and into the primary bronchus. In these reptiles, CT scans can offer precise information on the distribution of the disease for additional considerations, such as pulmonoscopy. In chelonians, CT scans can also help localize lesions to determine the best location to perform a surgical access window in the carapace to access the lungs. This would allow for pulmonoscopy to visualize the extent of the disease, obtain samples for histology and culture, and even perform intralesional therapy, particularly in cases of granulomas. If obtaining a CT scan is not possible, then radiographs would certainly be indicated. However, one must consider that a lack of observed pulmonary abnormalities is not enough to rule out respiratory diseases. It is also important to remember that terminology for pulmonary patterns used in mammals (i.e., alveolar pattern, bronchial pattern, etc.) cannot be applied to reptiles because the anatomy is different and most lack a bronchial tree. The trabeculae can be observed on radiographs (Figure 10), prompting a misdiagnosis of bronchial pattern. Perhaps the most useful information obtained from radiographs is an evaluation of the pulmonary distention. In the author’s experience, reptile lungs typically encompass 40–60% of the coelomic cavity regardless of the respiratory phase (Figure 11 and Figure 12). When the lungs encompass less than 40% of the coelomic cavity, the author advises further evaluation to differentiate pulmonary vs. extrapulmonary disease, such as a coelomic mass, distended intestinal tract, follicles, or eggs. Positioning is also important, as one study found that extension of the neck and limbs led to an increase in lung volume on CT images of a red-eared slider (*Trachemys scripta elegans*) [21].

An additional diagnostic tool is tracheal or pulmonary lavage. The distinction of whether a tracheal or pulmonary sample is obtained depends on the size of the patient, the anatomy of the lungs, and the length of the catheter utilized to obtain the sample. In smaller patients with unicameral or paucicameral lungs, it could be possible to infuse enough fluids to reach the lumen of the lungs. In larger animals such as snakes or those with multicameral lungs, especially chelonians and varanids, it would be unrealistic to expect much of that fluid to reach the parenchymal tissue. Therefore, in those animals, the sample will mostly be a tracheal or bronchial lavage. The lavage must be performed aseptically to minimize contamination from the oral cavity and ensure that any organisms identified are indeed of respiratory origin. Sterile gloves, lubricant, syringe, and catheter are used. The author uses sterile saline infused at a rate of 3% body weight for those with unicameral or transitional lungs. For those with multicameral lungs, the volume should be 1% of the body weight. In the author’s experience, one can expect to recover approximately 40–50% of the infused volume from species with unicameral or transitional lungs and 10–30% from those with multicameral lungs. For this reason, being more conservative in species with multicameral lungs may avoid any complications from excess fluid remaining in the lungs. This is critical in patients presenting with severe respiratory signs. With the patient positioned vertically with the head up towards the ceiling, the sterile catheter can be inserted into the trachea, being sure not to touch the oral mucosa to avoid contamination. Once the catheter is inserted, the full volume of saline is infused. After a brief period of approximately 5 s, the animal is inverted with the head down towards the ground, and gentle aspiration of the syringe connected to the catheter is performed to remove as much fluid as possible. In lizards and snakes, the coelom can be gently pressed to help drain fluid out of the lungs. A good sample will be characterized by being cloudier than the saline and often having some white-to-tan free-floating material. This sample can then be used for cytology, bacterial culture and sensitivity, fungal culture, and/or PCR for infectious diseases. Therapy can be selected based on presentation, clinical signs, and diagnostics.

In species with homogeneous faveolar lungs, it is expected that systemic therapeutic drugs will be able to reach this tissue more effectively, and hence, the prognosis for response to therapy would be better. However, in those with homogeneous edicular lungs or with heterogeneous lungs, systemic drugs are unlikely to effectively reach the parenchyma, hence rendering treatment less effective. In reptiles with edicular lungs, additional therapeutic routes such as nebulization should be considered. This ensures delivery of drugs directly to the parenchyma without reliance on venous supply. While nebulization is commonly used in a clinical setting, there is a need for additional studies on the effectiveness of this treatment method. Nebulized terbinafine reached therapeutic plasma concentrations in cottonmouth snakes (*Agkistrodon piscivorus*) [22]. In addition to a multimodal therapeutic approach, knowledge of the portion of the lung that may be affected aids in evaluating the expected response to therapy.

The lung anatomy of reptiles has clinical implications for the diagnosis and treatment of respiratory diseases. A plan must be formulated for each patient based on the species, lung anatomy, and severity of the clinical signs.

## 4. Physiology

Reptilian lungs are essential for functions beyond respiration. They also play a role in temperature regulation through evaporation, buoyancy (e.g., marine snakes and some aquatic chelonians), communication (e.g., crocodilian resonance), and defense (e.g., lung inflation to increase size, hissing, or lodging into a crevice). Reptile lungs have large volumes but only 10–20% of the surface area of mammalian lungs [10]. The residual volume is minimal in unicameral and transitional lungs [10]. The trabeculae, septa, and large amounts of surfactants prevent atelectasis. Reptile lungs also have higher compliance, making it easier to re-inflate, even after extended periods of breath holding [23]. The trabeculae have abundant smooth muscle. There are also neuroepithelial bodies, which, during periods of hypoxia, secrete serotonin to stimulate muscle contraction. This results in eversion of the trabeculae towards the lumen to increase surface area, allowing for more capillary exposure and improved gas exchange [24,25].

The respiratory system is intricately associated with the cardiovascular system in the process of right-to-left shunting. Gas exchange occurs by diffusion when oxygen-rich air contacts the pulmonary capillary blood supply [26]. There are various studies that have evaluated the movement of air in the respiratory tract of reptiles and show a unidirectional flow of air through the respiratory tract analogous to the air movement in birds. This unidirectional flow has been reported in green iguanas [27], savannah monitors (*Varanus exanthematicus*) [9], American alligators [28], and the Nile crocodile (*Crocodylus niloticus*) [6]. The caudal portion of the bronchi or lungs in these species serves as a reservoir of air and creates a predicted flow through the lungs. Some portions experience unidirectional flow while others have bidirectional flow at different phases of respiration. These findings may have significant clinical implications as it relates to calculating proper tidal volume and oxygen flow of anesthetized reptiles. It also has implications for the extent to which pulmonary disease may affect oxygenation, depending on which portion of the lung is affected. From a therapeutic standpoint, it illustrates that nebulized agents may not necessarily have an even distribution in the parenchyma. Further research is needed in each of these areas to bridge the gap between physiology and clinical medicine.

A ventilation cycle has three phases: expiration, inspiration, and relaxation phase (apnea phase). When the lungs are inflated, and the glottis is closed, contraction of the smooth muscle in the trabeculae leads to tensioning of the parenchyma and an equalization of the intrapulmonary pressure [10] during apnea. This allows the lungs to maintain their shape and volume but also increases intrapulmonary pressure. An additional effect is hypoxic pulmonary vasoconstriction, which adds to the pulmonary resistance, further reducing pulmonary blood flow. Hypoxic pulmonary vasoconstriction has been documented in savannah monitors, broad-snouted caiman, and Argentine black and white tegu (*Salvator merianae*) but not in South American rattlesnakes (*Crotalus durissus*) [29]. It was also documented in pond sliders (*Trachemys scripta*) [30]. This vasoconstriction is locally mediated and helps to maintain the ventilation and perfusion match. These mechanisms result in an increased pulmonary resistance, which stimulates right-to-left cardiac shunting. The net result is a decreased pulmonary blood flow with an increased systemic blood flow. Bradycardia is a third effect observed during right-to-left shunting. Therefore, we can summarize that when a reptile takes a breath and closes its glottis, it is, in essence, pressurizing the respiratory system. This leads to an increased pulmonary vascular resistance, a decrease in pulmonary blood flow, and bradycardia that results in cardiac right-to-left shunting [31]. This leads to a reduction in PO2 in the left and right aortas [31]. These are not the only factors that determine activation of the shunt, but they are the physical and anatomical drivers behind it. Temperature, hydration status, age, metabolic need, and other factors are bound to influence the need for oxygen exchange, directly influencing the degree and duration of shunting. During right-to-left shunting, less blood is delivered to the lungs, allowing a reduction in oxygen uptake. This, in turn, allows animals to remain in the apnea phase, whether it is for diving or remaining inflated to help avoid a predator’s attack. As time goes by and more oxygen is needed, the reptile can reduce the amount of shunting to allow for oxygen exchange. This system is driven by the need for and ability to extract oxygen from the respiratory system. Another tool to aid in this process was mentioned previously, by which trabecular smooth muscles are activated to expose the capillaries to the lumen of the lung [10]. This increases the surface area and hence maximizes oxygen uptake. Once the reptile can no longer extract oxygen from the accumulated air, it will be forced to enter the expiration phase, followed by inspiration to fill the lungs with fresh air once again. While both O_2_ and CO_2_ receptors are present in reptile lungs, it is the PO_2_ that seems to have a more significant impact on shunting and respiration. Hypoxia has been shown to have varied effects on ventilation through changes to the breathing frequency and tidal volume [32,33]. These changes are not equal or consistent across species, adding to the complexity of understanding the respiratory physiology of reptiles [32]. Positioning can also affect respiration. Yellow-bellied sliders (*Trachemys scripta scripta*) placed on dorsal recumbency were shown to have reduced pulmonary efficacy with altered arterial and alveolar oxygen concentrations suggestive of compromised gas exchange [34]. Intermittent positive pressure ventilation (IPPV) is needed in these patients to help overcome the impact of dorsal recumbency [34]. IPPV helps to create fluctuations in intrapulmonary pressure to help minimize the shunting stimulus. In addition, positioning patients with their head slightly elevated can help minimize pressure from the viscera resting on the lungs while they are on dorsal recumbency. Other factors, such as temperature and hydration, are also bound to influence the responses to hypoxia and hypercapnia. Nonetheless, at the most basic level, we must remember that reptiles can tolerate physiological extremes beyond those of mammals. If there is enough oxygen-rich air in the lungs and they are right-to-left shunting, they can remain in the apnea phase of ventilation for longer periods of time. In addition, the metabolic demand for oxygen will also have a significant impact on shunting, ventilation, and perfusion changes. One study evaluated the use of atropine to help overcome right-to-left shunting in red-foot tortoises (*Chelonoidis carbonaria*) under isoflurane [35]. The investigators were able to show that atropine administration reduced shunting and lowered the minimum anesthetic concentration of isoflurane [35]. These results are in line with reports of epinephrine administration leading to faster anesthetic recoveries in common snapping turtles (*Chelydra serpentina*) [36] and American alligators [37]. Both pharmacological approaches work to overcome shunting, but they may not be without possible complications. In rats, the intravenous administration of atropine led to a decrease in pulmonary gas exchange [38]. Epinephrine administration in rats led to hypoxemia, hypercapnia, and acidosis, leading the authors to question the doses commonly used for resuscitation [39]. In human infants, nebulized epinephrine was shown to improve pulmonary resistance but not oxygenation or ventilation [40]. Extrapolations of information from mammalian models to reptiles should be interpreted cautiously until similar studies can be carried out in reptiles. However, these mammalian studies should serve as a reminder that use of these drugs is not without possible consequences and, in fact, may deteriorate perfusion and oxygen uptake. Therefore, their use should be applied with extreme caution until we can better understand their effects on reptile respiratory physiology.

Cardiac shunting is a normal physiologic adaptation of reptiles. Therefore, the clinical goal is not to avoid shunting but rather to minimize its effects on reptiles under anesthesia. With a better understanding of the physiology of shunting, clinicians can make educated decisions about how to best manage individual patients. Historically, there has been a clinical focus on the speed of recovery of anesthetized reptiles. The reduction in inspired oxygen fraction from 100% to 21% (room air) near the end of anesthesia has been utilized as one of the tools to improve recovery. The theory of this rationale is that reptile lungs are primarily responsive to oxygen for ventilation. This has perhaps been a simplistic view of the complex physiological processes that occur in anesthetized reptiles. In chelonians, there is physiological data that supports the fact that hypoxia increases ventilation [32]. However, the ventilatory response of the Amazon lava lizard (*Tropidurus torquatus*) was shown to be more sensitive to changes in CO_2_ than O_2_ [41]. A seasonal variability to this response was also reported [41]. In Brazilian short-tailed boas (*Boa constrictor amaralis*) and South American rattlesnake, hypoxia and hypercarbia increased minute ventilation, but hypoxia had a lower effect [42]. They also observed that hypercarbia led to a decrease in gas exchange while hypoxia had no significant effect on this variable. In garter snakes (*Thamnophis sirtalis*), hypoxia increased tidal volume [43].

One important consideration is that most of these studies used either nitric oxide gas or levels of oxygen below 10% to induce hypoxia. This does not represent the clinical scenario of exposure to 21% oxygen. Veterinary studies have evaluated the effect of the inspired fraction of oxygen. These studies add to the knowledge of reptilian physiology but have yet to replicate the most common clinical application of maintenance at 100%, with a decrease to 21% at the end of the procedure. A study in green sea turtles (*Chelonia mydas*) compared delivery of sevoflurane in animals mechanically ventilated with 100% vs. 21% oxygen for 90 min [44]. Higher SpO_2_ was measured in the 100% oxygen group, but there were no differences in venous blood gases or time to recovery [44]. In common snapping turtles anesthetized with alfaxalone and supplemented with 100% vs. 21% oxygen, a higher venous partial pressure of oxygen was observed in the 100% oxygen group [45]. However, there were no differences in any other parameters, such as time to recovery [45]. In another study, bearded dragons were sedated with alfaxalone and administered 100% vs. 21% oxygen via face mask during spontaneous breathing for 100 min [46]. Venous Pco_2_ and HCO_3_^−^ were higher in the 21% oxygen group, but there were no clinically significant effects on outcomes. Another study on bearded dragons compared isoflurane delivered with 100% vs. 21% oxygen for 60 min [47]. The only statistical difference was a lower SpO_2_ in the 21% group. While not statistically significant, the authors pointed out that times to induction and recovery were overall shorter for the 21% group [47]. Green iguanas anesthetized with isoflurane had faster recoveries when ventilated with 21% vs. 100% oxygen [48]. However, all these studies compared 100% vs. 21% oxygen during the whole duration of the procedure. No studies have evaluated a change from maintenance at 100% oxygen followed by 21% during the recovery phase, which is the more common clinical application of reptile anesthesia.

Malte and Wang showed that achieving a steady state of gas exchange in response to hypercapnia and hypoxia could take over 300 min in Cuvier’s dwarf caiman (*Paleosuchus palpebrosus*) [49]. The clinical studies previously mentioned only evaluated animals anesthetized for 60 to 100 min. Therefore, additional studies are needed to determine if the time to reach a steady state of gas exchange in reptiles with three-chambered hearts would also be near or above 300 min. It is also likely that this impact may be different across species. This remains an important area for future research that will continue to revolutionize our knowledge of reptile physiology.

## 5. Conclusions

Reptiles have a varied and unique anatomy of the respiratory tract. Unique adaptations of the upper respiratory tract include vascular cavernous tissue in the nasal passages of crocodilians and the extension of the nasal opening in marine iguanas. The lower respiratory tract is even more complex with varied configurations of tracheal rings and the presence of gular pouches in chameleons. The lungs can be homogeneous or heterogeneous, faveolar or edicular, and some end in air sacs. Diagnostic evaluation of respiratory structures is best achieved with a CT scan to aid in determining the extent and localization of disease. Radiographs can also provide useful information, but are often a less sensitive test during acute stages of respiratory disease. Endoscopy can be used to target diagnostic sampling for additional tests such as biopsy, cultures, and PCR. Based on the lung anatomy and disease distribution, nebulization should be considered as an adjunct therapy to systemic drugs. Knowledge of the specific anatomy for each taxon and species is essential for the interpretation of diagnostics and selection of the best therapeutic approach.

The physiology of reptilian respiration is perhaps even more complex than their anatomy. Studies have revealed airflow patterns like those observed in avian species, even in relatively simple transitional lungs of green iguanas [27]. An added layer of complexity is the interconnection between the pulmonary and cardiac systems. Pulmonary pressure is one of the factors that stimulates right-to-left cardiac shunting. The unidirectional air flow, diffusion-based gas exchange, three phases of ventilation, and cardiac shunting create a complex physiological response with clinical implications for anesthesia of reptiles. Cardiac shunting is a normal physiologic adaptation of reptiles. Therefore, the clinical goal is not to avoid shunting but rather to minimize its effects on reptiles under anesthesia. With a better understanding of the physiology of shunting, clinicians can make educated decisions about how to best manage individual patients. Recent studies have evaluated the effects of inspired fractions of oxygen and drugs like atropine and epinephrine on recovery from anesthesia. Many of these studies show promise for improvement in anesthetic events. However, the possible deleterious effects of trying to overcome cardiac shunting through pharmacological means should be investigated to ensure they are indeed safe.

Ultimately, the information presented should highlight the fact that reptiles are not a monolithic group of animals with equal anatomy and physiology across taxa. Therefore, a paradigm shift is needed to start thinking of the anatomy and physiology of individual species rather than believing that a single concept applies to all reptiles equally. As such, future research should aim to evaluate these concepts across the more common species encountered in clinical practice.

## Data Availability

Not applicable.

## References

[1] Klassen M., Adams J., Cramberg M., Knoche L., Young B.A. (2020). The narial musculature of *Alligator mississippiensis*: Can a muscle be its own antagonist?. J. Morphol..

[2] Miller L.R., Gutzke W.H. (1999). The role of the vomeronasal organ of crotalines (Reptilia: Serpentes: Viperidae) in predator detection. Anim. Behav..

[3] Lima M.O., Gorza L.L., Borges E.J.S., Paula V.T., Nunes L.C., Nóbrega Y.C., Figueiredo R.G., Silva M.A.D. (2024). Morphological comparison of the larynx and trachea of *Chelonia mydas* (Linnaeus, 1758), *Caiman yacare* (Daudin, 1802) and *Caiman latirostris* (Daudin, 1802). An. Acad. Bras. Cienc..

[4] Habova M., Pyszko M., Horak O., Cermakova E., Paral V. (2022). Differences in the anatomy of the lower respiratory tract in selected species of the order Testudines. Vet. Med..

[5] Klingler J.J. (2016). On the morphological description of tracheal and esophageal displacement and its phylogenetic distribution in Avialae. PLoS ONE.

[6] Schachner E.R., Hutchinson J.R., Farmer C. (2013). Pulmonary anatomy in the Nile crocodile and the evolution of unidirectional airflow in Archosauria. PeerJ.

[7] Huskey S., Tegge S.M., Anderson C.V., Smith M.E., Barnett K. (2020). Gular pouch diversity in the Chamaeleonidae. Anat. Rec..

[8] Lyson T.R., Schachner E.R., Botha-Brink J., Scheyer T.M., Lambertz M., Bever G.S., Rubidge B.S., de Queiroz K. (2014). Origin of the unique ventilatory apparatus of turtles. Nat. Commun..

[9] Schachner E.R., Cieri R.L., Butler J.P., Farmer C.G. (2014). Unidirectional pulmonary airflow patterns in the savannah monitor lizard. Nature.

[10] Perry S.F. (1983). Reptilian lungs. Functional anatomy and evolution. Adv. Anat. Embryol. Cell Biol..

[11] Perry S.F. (1998). Lungs: Comparative anatomy, functional morphology, and evolution. Biol. Reptil..

[12] Peixoto D., Klein W., Abe A., da Cruz A. (2018). Functional morphology of the lungs of the green iguana, *Iguana iguana*, in relation of Body mass (Squamata: Reptilia). Vertebr. Zool..

[13] Daniels C.B., McGregor L.K., Nicholas T.E. (1994). The dragon’s breath: A model for the dynamics of breathing and faveolar ventilation in agamid lizards. Herpetologica.

[14] Lillywhite H.B., Albert J.S., Sheehy C.M., Seymour R.S. (2012). Gravity and the evolution of cardiopulmonary morphology in snakes. Comp. Biochem. Physiol. A Mol. Integr. Physiol..

[15] Lillywhite H.B. (2025). Pulmonary structure and function in marine snakes. Zool. Sci..

[16] Klein W., Owerkowicz T. (2006). Function of intracoelomic septa in lung ventilation of amniotes: Lessons from lizards. Physiol. Biochem. Zool..

[17] Klaver C.H. (1973). Lung anatomy: Aid in Chameleon taxonomy. Beaufortia.

[18] Uriona T.J., Lyon M., Farmer C.G. (2009). The importance of the *M. diaphragmaticus* to the duration of dives in the American alligator (*Alligator mississippiensis*). Zoology.

[19] Stockman J., Innis C.J., Solano M., O’Sullivan Brisson J., Kass P.H., Tlusty M.F., Weber E.S. (2013). Prevalence, distribution, and progression of radiographic abnormalities in the lungs of cold-stunned Kemp’s ridley sea turtles (*Lepidochelys kempii*): 89 cases (2002–2005). J. Am. Vet. Med. Assoc..

[20] Pees M., Kiefer I., Oechtering G., Krautwald-Junghanns M.E. (2008). Computed tomography for the diagnosis and treatment monitoring of bacterial pneumonia in Indian pythons (*Python molurus*). Vet. Rec..

[21] Mans C., Drees R., Sladky K.K., Hatt J.M., Kircher P.R. (2013). Effects of body position and extension of the neck and extremities on lung volume measured via computed tomography in red-eared slider turtles (*Trachemys scripta elegans*). J. Am. Vet. Med. Assoc..

[22] Kane L.P., Allender M.C., Archer G., Leister K., Rzadkowska M., Boers K., Souza M., Cox S. (2017). Pharmacokinetics of nebulized and subcutaneously implanted terbinafine in cottonmouths (*Agkistrodon piscivorus*). J. Vet. Pharmacol. Ther..

[23] Hedrick M.S., Kohl Z.F., Khan M., Eme J., Dzialowski E.M., Crossley D.A. (2013). Lung volume and pulmonary compliance in the alligator (*Alligator mississippiensis*). FASEB J..

[24] van den Steen P., van Lommel A., Lauweryns J.M. (1994). Neuroepithelial bodies in the lung of *Basiliscus vittatus* (Reptilia, Iguanidae). Anat. Rec..

[25] Scheuermann D.W., De Groodt-Lasseel M.H., Stilman C., Meisters M.L. (1983). A correlative light-, fluorescence- and electron-microscopic study of neuroepithelial bodies in the lung of the red-eared turtle, *Pseudemys scripta elegans*. Cell Tissue Res..

[26] Wang T. (2011). Gas exchange in frogs and turtles: How ectothermic vertebrates contributed to solving the controversy of pulmonary oxygen secretion. Acta Physiol..

[27] Cieri R.L., Craven B.A., Schachner E.R., Farmer C.G. (2014). New insight into the evolution of the vertebrate respiratory system and the discovery of unidirectional airflow in iguana lungs. Proc. Natl. Acad. Sci. USA.

[28] Farmer C.G., Sanders K. (2010). Unidirectional airflow in the lungs of alligators. Science.

[29] Skovgaard N., Abe A.S., Andrade D.V., Wang T. (2005). Hypoxic pulmonary vasoconstriction in reptiles: A comparative study of four species with different lung structures and pulmonary blood pressures. Am. J. Physiol. Regul. Integr. Comp. Physiol..

[30] Crossley D., Altimiras J., Wang T. (1998). Hypoxia elicits an increase in pulmonary vasculature resistance in anaesthetised turtles (*Trachemys scripta*). J. Exp. Biol..

[31] Hicks J., Comeau S. (1994). Vagal regulation of intracardiac shunting in the turtle *Pseudemys scripta*. J. Exp. Biol..

[32] Johnson S.M., Krisp A.R., Bartman M.E. (2015). Hypoxia switches episodic breathing to singlet breathing in red-eared slider turtles (*Trachemys scripta*) via a tropisetron-sensitive mechanism. Respir. Physiol. Neurobiol..

[33] Abe A.S., Johansen K. (1987). Gas exchange and ventilatory responses to hypoxia and hypercapnia in *Amphisbaena alba* (Reptilia: Amphisbaenia). J. Exp. Biol..

[34] Williams C.J.A., Hansen K., Williams N., Jakobsen S.R., Pedersen C.C.E., Bertelsen M.F., Wang T. (2021). The influence of assisted ventilation and recumbency on cardiorespiratory physiology in the anesthetized freshwater turtle *Trachemys scripta scripta*. Comp. Biochem. Physiol. A Mol. Integr. Physiol..

[35] Greunz E.M., Williams C., Ringgaard S., Hansen K., Wang T., Bertelsen M.F. (2018). Elimination of intracardiac shunting provides stable gas anesthesia in tortoises. Sci. Rep..

[36] Goe A., Shmalberg J., Gatson B., Bartolini P., Curtiss J., Wellehan J. (2016). Epinephrine or GV-26 electrical stimulation reduces inhalant anesthestic recovery time in common snapping turtles (*Chelydra serpentina*). J. Zoo Wildl. Med..

[37] Gatson B.J., Goe A., Granone T.D., Wellehan J.F. (2017). Intramuscular epinephrine results in reduced anesthetic recovery time in American alligators (*Alligator mississippiensis*) undergoing isoflurane anesthesia. J. Zoo Wildl. Med..

[38] Gaspari R.J., Paydarfar D. (2014). Pulmonary effects of intravenous atropine induce ventilation perfusion mismatch. Can. J. Physiol. Pharmacol..

[39] Krishnamoorthy V., Hiller D.B., Ripper R., Lin B., Vogel S.M., Feinstein D.L., Oswald S., Rothschild L., Hensel P., Rubinstein I. (2012). Epinephrine induces rapid deterioration in pulmonary oxygen exchange in intact, anesthetized rats: A flow and pulmonary capillary pressure-dependent phenomenon. Anesthesiology.

[40] Numa A.H., Williams G.D., Dakin C.J. (2001). The effect of nebulized epinephrine on respiratory mechanics and gas exchange in bronchiolitis. Am. J. Respir. Crit. Care Med..

[41] Longhini L.S., Porto L.S., Rocha A.C.G., Bícego K.C., Klein W., Gargaglioni L.H. (2019). Seasonal variation of hypoxic and hypercarbic ventilatory responses in the lizard *Tropidurus torquatus*. Comp. Biochem. Physiol. A Mol. Integr. Physiol..

[42] Oda G.M., Leite C.A.C., Abe A.S., Klein W. (2021). Effects of different levels of hypoxia and hypercarbia on ventilation and gas exchange in *Boa constrictor amaralis* and *Crotalus durissus* (Squamata: Serpentes). Respir. Physiol. Neurobiol..

[43] Bartlett D., Birchard G.F. (1983). Effects of hypoxia on lung volume in the garter snake. Respir. Physiol..

[44] Justo A.A., Dutra G.H.P., Carregaro A.B., Cortopassi S.R.G. (2023). The fraction of inspired oxygen does not affect the time to extubation in mechanically ventilated, sevoflurane-anesthetized green sea turtles (*Chelonia mydas*). Am. J. Vet. Res..

[45] Martony M.E., McMahon S., Bailey J., Bosworth L., Thompson L., Balko J.A. (2026). Evaluation of 100% versus 21% oxygen supplementation in common snapping turtles (*Chelydra serpentina*) anesthetized with alfaxalone. J. Zoo Wildl. Med..

[46] Ratliff C., Parkinson L.A.B., Mans C. (2019). Effects of the fraction of inspired oxygen on alfaxalone-sedated inland bearded dragons (*Pogona vitticeps*). Am. J. Vet. Res..

[47] Odette O., Churgin S.M., Sladky K.K., Smith L.J. (2015). Anesthetic induction and recovery parameters in bearded dragons (*Pogona vitticeps*): Comparison of isoflurane delivered in 100% oxygen versus 21% oxygen. J. Zoo Wildl. Med..

[48] Diethelm G., Mader D. (1999). The effects of FIO2 on post anesthetic recovery times in the green iguana. Proceedings of the Association of Reptilian and Amphibian Veterinarians.

[49] Malte C.L., Malte H., Wang T. (2016). The long road to steady state in gas exchange: Metabolic and ventilatory responses to hypercapnia and hypoxia in Cuvier’s dwarf caiman. J. Exp. Biol..

